# Selecting serum-free hepatocyte cryopreservation stage and storage temperature for the application of an “off-the-shelf” bioartificial liver system

**DOI:** 10.1038/s41598-024-60711-5

**Published:** 2024-05-28

**Authors:** Ji-Hyun Lee, Hey-Jung Park, Young-A Kim, Doo-Hoon Lee, Jeong-Kwon Noh, Jong-Gab Jung, Mal Sook Yang, Jong Eun Lee, Se Hoon Lee, Hee-Hoon Yoon, Suk-Koo Lee, Sanghoon Lee

**Affiliations:** 1 Stem Cell and Regenerative Medicine Center, Research Institute for Future Medicine, Samsung Medical Center, Seoul, Republic of Korea; 2 Research Institute, HLB Cell, Co. Ltd., Hwaseong, Republic of Korea; 3 Department of Surgery, School of Medicine, Samsung Medical Center, Sungkyunkwan University, 50 Irwon-Dong, Gangnam-Gu, Seoul, 06354 Republic of Korea; 4 Department of Surgery, Myongji Hospital, Goyang, Republic of Korea

**Keywords:** Biotechnology, Gastroenterology

## Abstract

The bioartificial liver (BAL) system can potentially rescue acute liver failure (ALF) patients by providing partial liver function until a suitable donor liver can be found or the native liver has self-regenerated. In this study, we established a suitable cryopreservation process for the development of an off-the-shelf BAL system. The viability of hepatocyte spheroids cryopreserved in liquid nitrogen was comparable to that of fresh primary hepatocyte spheroids. When hepatocyte spheroids were subjected to cryopreservation in a deep freezer, no statistically significant differences were observed in ammonia removal rate or urea secretion rate based on the cryopreservation period. However, the functional activity of the liver post-cryopreservation in a deep freezer was significantly lower than that observed following liquid nitrogen cryopreservation. Moreover, cryopreserving spheroid hydrogel beads in a deep freezer resulted in a significant decrease (approximately 30%) in both ammonia removal and urea secretion rates compared to the group cryopreserved in liquid nitrogen. The viabilities of spheroid hydrogel beads filled into the bioreactor of a BAL system were similar across all four groups. However, upon operating the BAL system for 24 h, the liver function activity was significantly higher in the group comprising hydrogel beads generated after thawing hepatocyte spheroids cryopreserved in liquid nitrogen. Consequently, the manufacturing of beads after the cryopreservation of hepatocyte spheroids is deemed the most suitable method, considering efficiency, economic feasibility, and liver function activity, for producing a BAL system.

## Introduction

Acute liver failure (ALF) is a fatal disease with a mortality rate of 80–90% without liver transplantation (LT). Even with the application of LT for treatment of ALF the mortality rate exceeds 50%^1,2^. The complex function of the liver makes management of ALF especially challenging as it not only detoxifies toxic byproducts but also participates in numerous synthetic and metabolic functions of the body. Bioartificial liver (BAL) support systems have been developed with the goal of augmenting the deteriorating liver function in ALF patients until a suitable donor liver can be found for LT or the native liver has regenerated^3^. BAL systems include a bioreactor filled with hepatocytes that provide detoxification and biosynthetic liver functions^4^. Several hollow fiber-based BAL devices have been developed and undergone clinical trials to analyze its efficacy with varying degrees of clinically relevant outcomes^5–7^. However, given the rapidly progressive nature of ALF and the adverse patient outcomes associated with delays in management, the timely production of a well-functioning bioreactor with readily accessible cell sources is imperative for the successful clinical implementation of a BAL system in ALF management.

Production of a BAL system for clinical application begins with the isolation of fresh hepatocytes from source animals. Subsequently, a bioreactor housing high-purity hepatocytes is constructed and rigorous post-production safety and efficacy tests ensue, ensuring the system’s reliability. Once validated, the BAL system is ready for patient application. The entire process of obtaining a clinically applicable BAL system is time-consuming, which poses a significant challenge to its successful commercialization^8^. Cryopreserved hepatocyte spheroids can be rapidly thawed and integrated into the BAL system for treating patients with acute liver failure requiring emergent care. This approach not only reduces the time spent on transporting animals, isolating hepatocytes, and culturing spheroids but also enhances safety by allowing pre-testing for infectious pathogens.

In this study we compared hepatocyte spheroids and spheroids hydrogel beads as targets for cryopreservation in the manufacturing process of a readily available “off-the-shelf” BAL system. In order to select the optimal cryopreservation steps and procedures, we created four groups of spheroids hydrogel beads for filling in a bioreactor that is 1/10 the size of the actual BAL: (1) SLNB, hydrogel beads prepared by cryopreserving hepatocyte spheroids in liquid nitrogen and then thawing them, (2) SDFB, hydrogel beads prepared by cryopreserving hepatocyte spheroids in a deep freezer and then thawing them, (3) BLN, hepatocyte spheroids were immobilized with hydrogel beads and then cryopreserved in liquid nitrogen, and (4) BDF, hepatocyte spheroids were immobilized with hydrogel beads and then cryopreserved in a deep freezer. These spheroid hydrogel beads were applied to the BAL system and operated for 24 h to compare and analyze hepatocyte metabolism.

## Results

### Viability and liver-specific functions of cryopreserved hepatocyte spheroids

**Figure 1:**
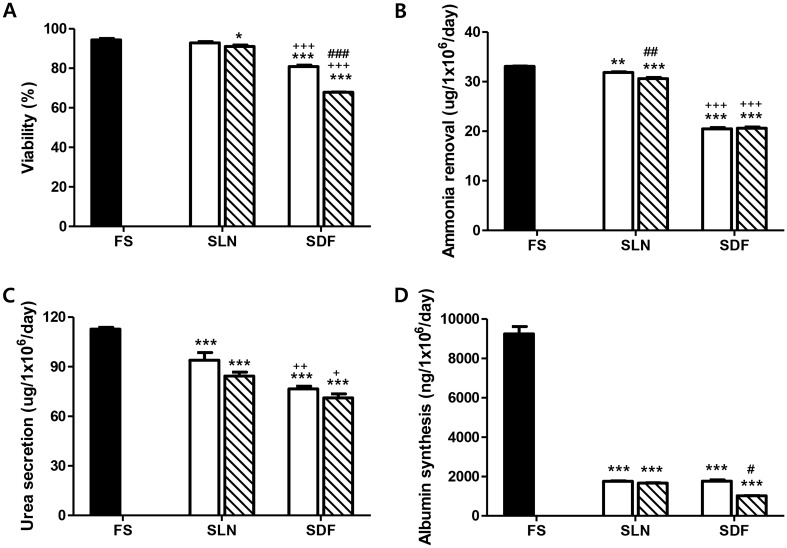
Effects of cryopreservation temperature and duration on hepatocyte spheroids’ viability and liver-specific functions in post-thaw cultures. Porcine hepatocyte spheroids were cryopreserved in a cryopreservation solution and stored for 1 month (white bars) and 1 year (pattern bars) in liquid nitrogen (SLN) or a deep freezer (SDF). (**A**) Cell viability, (**B**) ammonia removal, (**C**) urea secretion, and (**D**) albumin synthesis levels were evaluated after cells were thawed, plated, and cultured for 24 h. Statistical significance compared to fresh primary hepatocyte spheroids (FS, black bar): *p < 0.05; **p < 0.01; ***p < 0.001. Statistical significance comparing SLN to SDF: ^+^p < 0.05; ^++^p < 0.01; ^+++^p < 0.001. Statistical significance comparing 1 month (white bars) to 1 year (pattern bars): #p < 0.05; ##p < 0.01; ###p < 0.001.

The viability of hepatocyte spheroids cryopreserved in liquid nitrogen (SLN) demonstrated similarity to that of fresh primary hepatocyte spheroids (FS) (Fig. 1A). However, hepatocyte spheroids cryopreserved in a deep freezer (SDF) exhibited significantly lower viability than FS. Notably, the viability of SLN did not exhibit significant differences between 1-month and 1-year cryopreservation periods, whereas SDF viability was significantly lower than that of FS (p < 0.001).

In Fig. 1B, it is evident that SDF exhibited a significantly lower ammonia removal rate than SLN (p < 0.001). The urea secretion rate was also notably lower in the SDF group than in the SLN group (p < 0.01) (Fig. 1C). Furthermore, the albumin secretion rate was significantly lower in the SDF group than in the FS group, but albumin secretion of SDF was not different from that in SLN (Fig. 1D).

### Viability and liver-specific functions of cryopreserved hepatocyte spheroid beads

**Figure 2:**
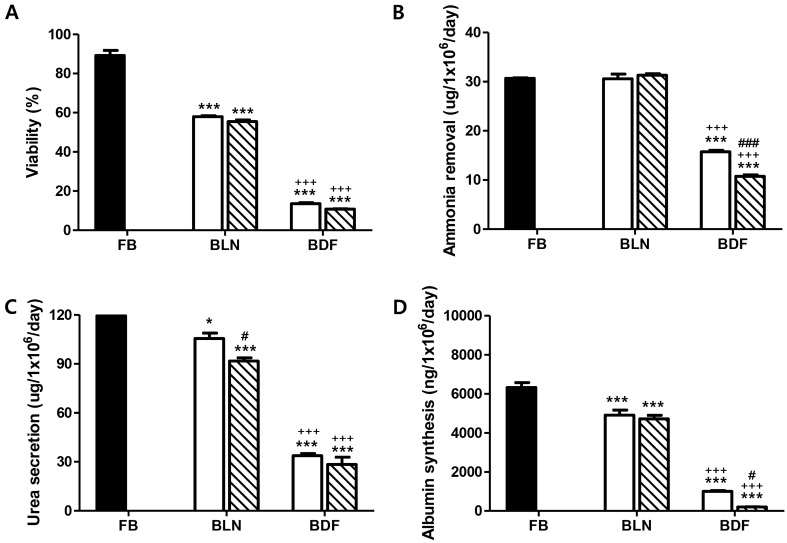
Effects of cryopreservation temperature and duration on hepatocyte spheroids beads’ viability and liver-specific functions in post-thaw cultures. Porcine hepatocyte spheroids beads were cryopreserved in a cryopreservation solution and stored for 1 month (white bars) or 1 year (pattern bars) in liquid nitrogen (BLN) or a deep freezer (BDF). (**A**) Cell viability, (**B**) ammonia removal, (**C**) urea secretion, and (**D**) albumin synthesis levels were evaluated after cells were thawed, plated, and cultured for 24 h. Statistical significance compared to fresh primary hepatocyte spheroid beads (FB, black bar): *p < 0.05; **p < 0.01; ***p < 0.001. Statistical significance comparing BLN to BDF: ^+^p < 0.05; ^++^p < 0.01; ^+++^p < 0.001. Statistical significance comparing 1 month (white bars) to 1 year (pattern bars): ^#^p < 0.05; ^##^p < 0.01; and ^###^p < 0.001.

Hepatocyte spheroid beads cryopreserved in liquid nitrogen (BLN) and in a deep freezer (BDF) exhibited significantly lower viabilities than those of fresh primary hepatocyte spheroid beads (FB) (Fig. 2A). Particularly, the BDF group exhibited a significantly lower survival rate than the BLN group (p < 0.001).

As illustrated in Fig. 2B, the BLN and FB groups demonstrated similar ammonia removal rates, whereas the BDF group displayed a progressively lower ammonia removal rate depending on the cryopreservation period (1 month and 1 year) (p < 0.001). Moreover, the rate of urea secretion was significantly lower in the BDF group than in the BLN group (p < 0.001) (Fig. 2C). All cryopreserved groups exhibited significantly lower albumin secretion rates than the FB group. Moreover, it was significantly lower in the BDF group than in the BLN group (p < 0.001) (Fig. 2D).

### Cryopreservation of hepatocyte spheroids and immobilized spheroid hydrogel beads using large-scale freezing bags

**Figure 3:**
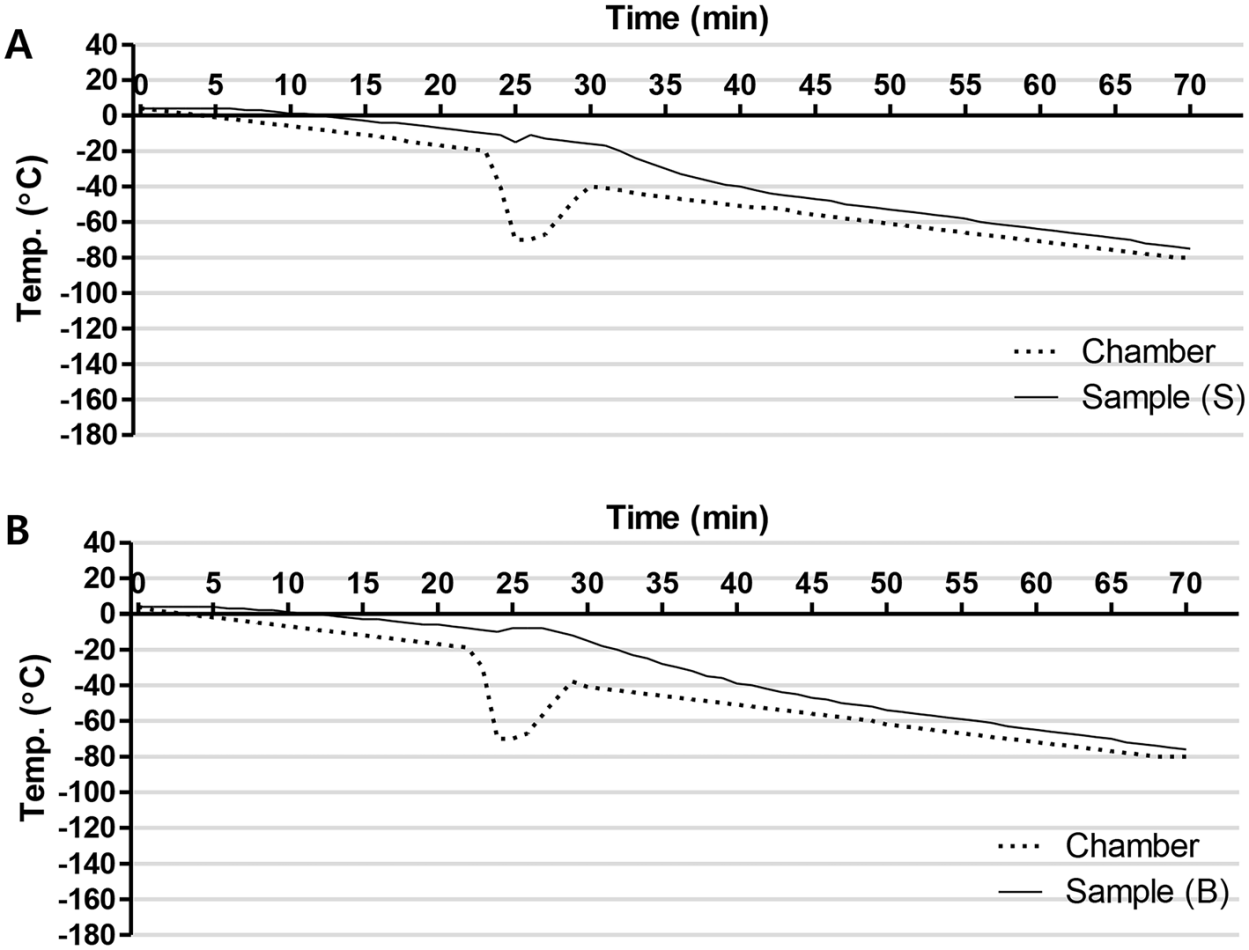
Comparison of the time–temperature profiles for freezing bags and chamber during cooling. Hepatocyte spheroids (**A**) and immobilized hepatocyte spheroids beads (**B**) were cooled by using an optimized cooling program. Traces show temperature of controlled rate freezer chamber (dotted black line) and the sample (solid black line). Sample (S): Hepatocyte spheroids bag; Sample (B), immobilized hepatocyte spheroids beads bag.

Using an optimized cooling program for the developed cryopreservation solution, the hepatocyte spheroids and hepatocyte spheroid hydrogel bead bags were subjected to freezing. Following this, a temperature–time history graph output was obtained (Fig. 3). The temperature exhibited a linear drop without an increase due to the latent heat of fusion.

### Analysis of hepatocyte spheroid cryopreservation scheme

**Figure 4:**
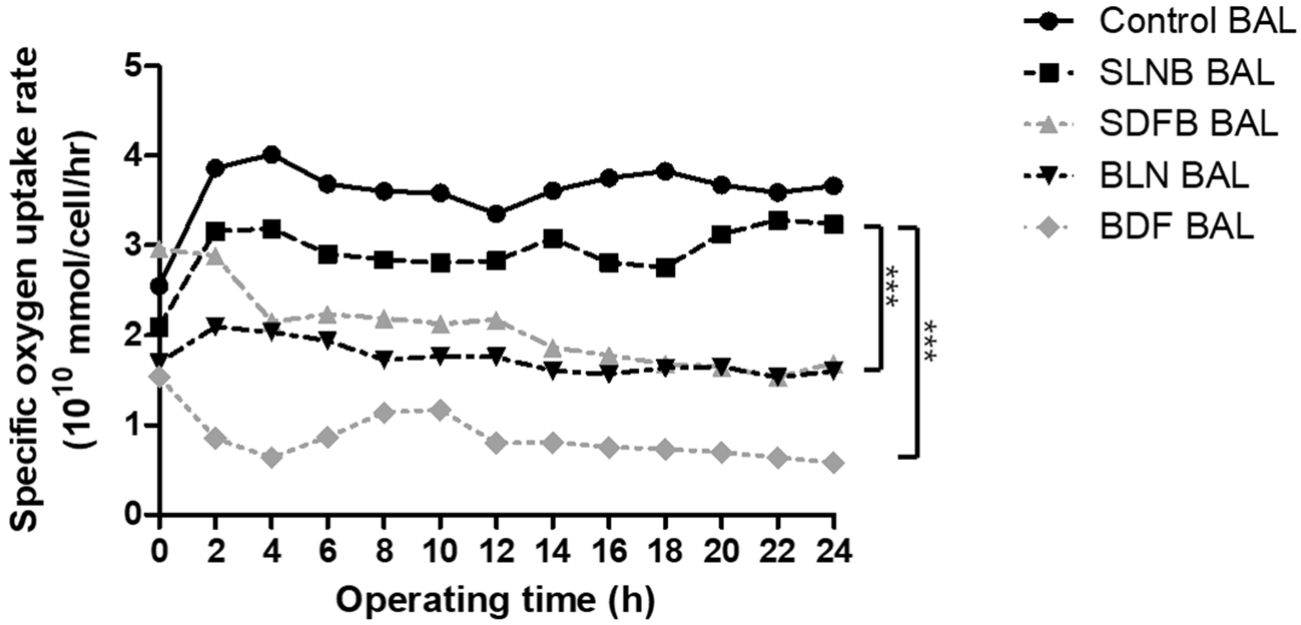
Specific oxygen uptake rate of immobilized hepatocyte spheroid in each group. A gas consisting of 95% oxygen and 5% carbon dioxide was supplied immediately upstream from the reactor. Data of dissolved oxygen were measured in real-time from samples collected from the circuit before and after the reactor in a bioartificial liver (BAL) system. The flow rate of the medium in the reactor was 30 mL/min. SLNB, LN_2_ freeze for 2 months/thaw–spheroids immobilization on hydrogel beads; SDFB, Deep freezer freeze for 2 months/thaw–spheroids immobilization on hydrogel beads; BLN, Spheroids immobilization on hydrogel beads–LN_2_ freeze for 2 months/thaw; BDF, Spheroids immobilization on hydrogel beads–deep freezer freeze for 2 months/thaw; Control, Spheroids immobilization on hydrogel beads–without freeze/thaw group. Specific oxygen uptake rates in BAL system after 24-h operation were analyzed by means of a two-way ANOVA and Bonferroni’s post multiple comparisons tests. ***p < 0.001.

We operated a 1/10 scaled-down version of the BAL system containing immobilized hepatocyte spheroid beads, produced based on each of the four freeze/thaw processes for 24 h (N = 5 in each group). The medium passes through the BAL system and recirculates through the reservoir, oxygenator/heater, and bioreactor containing hepatocyte spheroids hydrogel beads. We measured the dissolved oxygen levels within circulating media before and after the media entered the bioreactor. The functional performance of the bioreactor was evaluated according to the specific rate of oxygen uptake of hepatocyte spheroids (Fig. 4). In SLNB and control groups, an initial increase in specific oxygen uptake rate was observed, plateauing at 2–3 h. These rates were maintained throughout the 24-h operation in both groups. During 24 h of operation, specific oxygen uptake rates gradually decreased in the SDFB, BLN, and BDF groups. The SLNB group exhibited significantly higher uptake rates than the remaining three groups (p < 0.001). After 18 h operation, the specific rate of oxygen uptake in the SDFB and BLN groups was maintained at similar rates. However, throughout the operating period, all four study groups exhibited significantly lower specific oxygen uptake rates than the non-cryopreserved control group.

**Figure 5:**
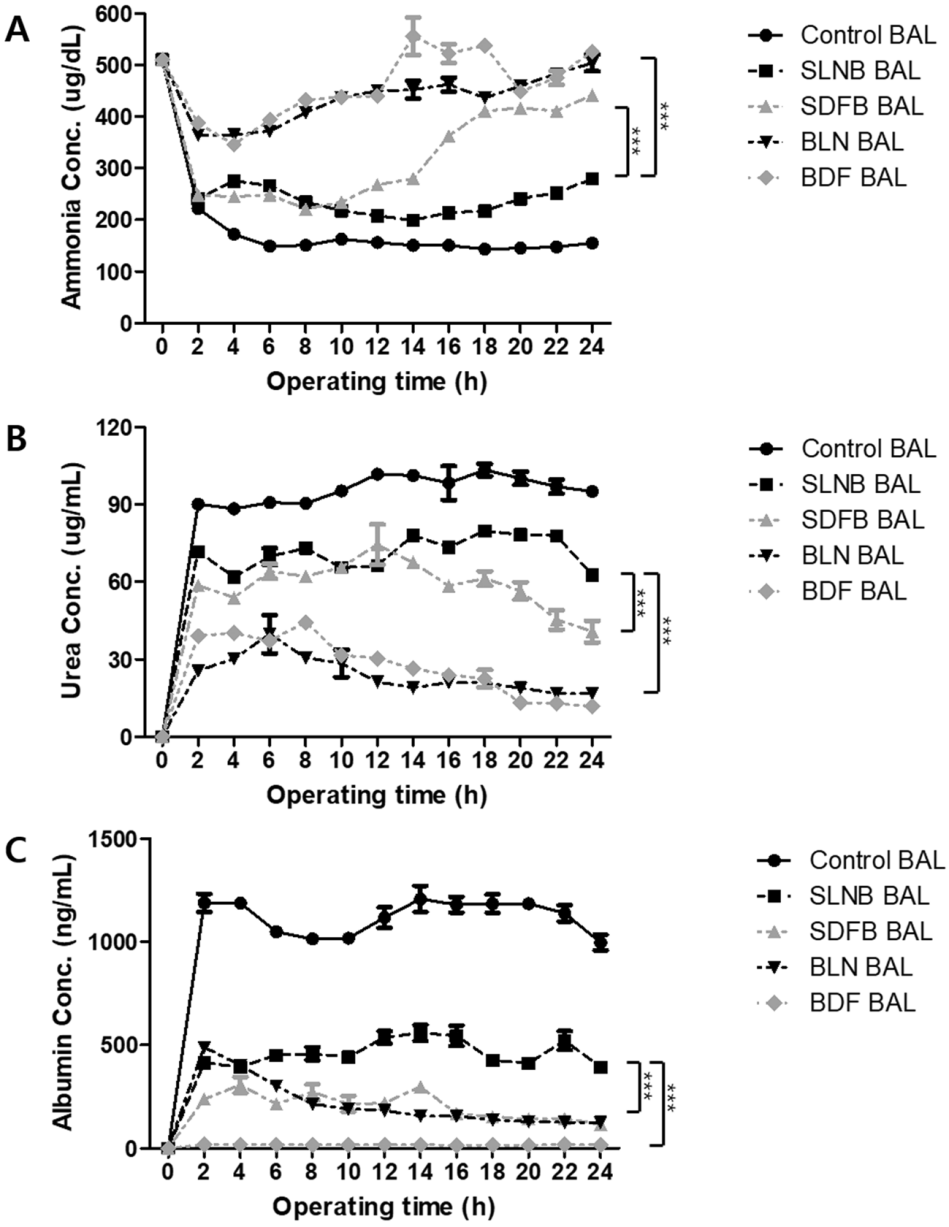
Functions of ammonia clearance, urea synthesis, and albumin synthesis in a bioartificial liver (BAL) system during 24-h operation. SLNB, LN_2_ freeze for 2 months/thaw–spheroids immobilization on hydrogel beads; SDFB, Deep freezer freeze for 2 months/thaw–spheroids immobilization on hydrogel beads; BLN, Spheroids immobilization on hydrogel beads–LN_2_ freeze for 2 months/thaw; BDF, Spheroids immobilization on hydrogel beads–deep freezer freeze for 2 months/thaw; Control, Spheroids immobilization on hydrogel beads–without freeze/thaw group. A two-way ANOVA and Bonferroni’s post multiple comparisons tests were performed to compare groups exposed to different freeze/thaw conditions in BAL system after 24-h operation. ***p < 0.001.

The assessment of ammonia removal by hepatocytes involved measuring ammonia concentrations in the outgoing media. Initially, ammonia concentration decreased, remaining stable throughout the BAL operation in the SLNB group and control group (Fig. 5A). However, ammonia concentration gradually increased during the 24-h operation in the SDFB, BLN, and BDF groups until approximately 10 h, after which ammonia in the SLNB and SDFB groups was maintained at similar concentrations. Subsequently, the ammonia concentration in the SDFB group increased. Notably, the SLNB group exhibited significantly lower ammonia levels than the other three groups (p < 0.001) after 10 h operation. Despite this, throughout the operating period, the four study groups exhibited significantly higher ammonia concentrations than the control group.

The levels of urea production and albumin in the outgoing media were evaluated. Initially, both urea and albumin concentrations increased, stabilizing during BAL operation in the SLNB and control groups (Fig. 5B,C). However, urea production gradually decreased during the 24-h operation in the SDFB, BLN, and BDF groups. At approximately 12 h, urea in the SLNB and SDFB groups was maintained at similar or slightly increased concentrations. However, urea concentrations decreased in the SDFB group following 12 h of BAL operation. Additionally, albumin production gradually decreased during the 24-h operation in the SDFB and BLN groups, with almost no albumin secretion in the BDF group. Compared to the other three groups, the SLNB group exhibited significantly higher levels of urea (p < 0.001) and albumin (p < 0.001) after 12 h operation. Nevertheless, all four study groups exhibited significantly lower urea and albumin levels than the control group.

**Figure 6:**
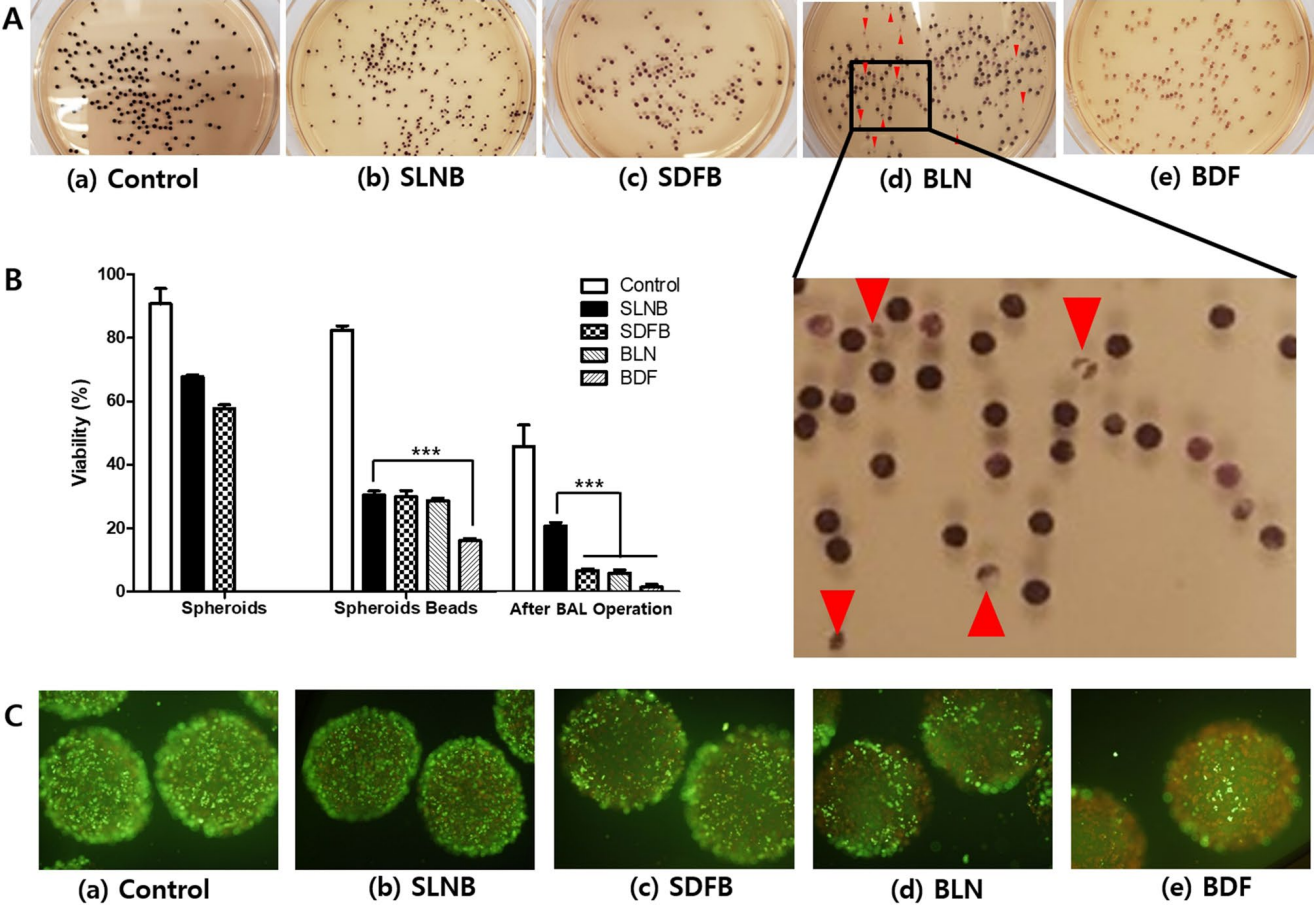
Photograph of each group's hepatocyte spheroids beads after the MTT assay before bioartificial liver (BAL) system operation (**A**), and viabilities of spheroids and spheroids beads in each step for BAL system operation (**B**). Representative images of porcine hepatocyte spheroids beads (**C**) showing their vital staining after BAL operation for 24 h. Live/Dead™ cell viability assay showing live cells stained with Calcein-AM (green) and dead cells stained with EthD-1 (red). SLNB, LN_2_ freeze for 2 months/thaw–spheroids immobilization on hydrogel beads; SDFB, Deep freezer freeze for 2 months/thaw–spheroids immobilization on hydrogel beads; BLN, Spheroids immobilization on hydrogel beads–LN_2_ freeze for 2 months/thaw; BDF, Spheroids immobilization on hydrogel beads–deep freezer freeze for 2 months/thaw; Control, Spheroids immobilization on hydrogel beads–without freeze/thaw group. The viability of hepatocyte spheroids and spheroids beads in BAL manufacturing/operation steps were analyzed by means of one-way ANOVA with Dunnett’s multiple comparison test. Statistical significance compared to SLNB group in spheroids bead step and after BAL operation: ***p < 0.001.

Figure 6A displays the hepatocyte spheroid beads following manufacturing or freezing/thawing along with the MTT assay. Although some beads appeared to be broken (Fig. 6A (d) red arrows), the viability was slightly higher following cryopreservation in liquid nitrogen (SLNB, BLN) than after cryopreservation in a deep freezer (SDFB, BDF). Bead breakage did not occur during cryopreservation in a deep freezer, and this result was consistent in several additional tests.

Figure 6B presents results analyzing the viabilities of hepatocyte spheroids and spheroid beads before BAL operation and the viability following 24 h of operation of each BAL system using the MTT assay. All five groups including the control group, exhibited significantly decreased viability after BAL operation. The viability of immobilized spheroid beads in the SLNB group was not significantly different from that of the SDFB and BLN groups but was significantly higher than that of the BDF group (p < 0.001). The viability of beads after 24 h of BAL operation was significantly higher in the SLNB group compared to the other groups (p < 0.001, control group excluded).

The viability of the hepatocyte spheroid beads after BAL operation for 24 h was represented using Live/Dead™ cell viability assay displaying live cells stained with Calcein-AM (green) and dead cells stained with EthD-1 (red) in Fig. 6C. The results of the MTT assay (Fig. 6B) were consistent with those obtained from the staining experiments (Fig. 6C).

## Discussion

The BAL system developed in this study uses a bioreactor filled with hepatocyte spheroid hydrogel beads. The purpose of this study was to establish an appropriate cryopreservation process for the development of an off-the-shelf BAL system. An essential consideration in the cryopreservation process is the temperature at which hepatocytes are preserved. The study compared the stability of long-term cryopreservation at liquid nitrogen (− 196 °C) and in a deep freezer (− 80 °C) temperatures, taking into account the associated costs for cryopreservation and transportation. Additionally, to consider the economic aspects of the BAL system manufacturing process, we compared and evaluated the spheroids beads manufacturing after the cryopreservation of hepatocyte spheroids and the cryopreservation process after manufacturing hepatocyte spheroids beads.

Previous studies on the cryopreservation of encapsulated liver spheroids (ELS) highlighted the importance of temperature. Cryopreservation at liquid nitrogen temperature for at least 1 year showed no significant loss of function^9^, whereas storage at − 196 °C followed by 8 days at − 80 °C resulted in substantial damage to ELS compared to direct thawing with liquid nitrogen^10^.

In the present study, hepatocyte spheroids cryopreserved in a deep freezer at − 80 °C exhibited no statistically significant difference in ammonia removal rate or urea secretion rate, irrespective of the cryopreservation period of 1 month or 1 year. However, the liver function activity in hepatocyte spheroids cryopreserved at − 80 °C was significantly lower than that in the group cryopreserved in liquid nitrogen. Cryopreserving hepatocyte spheroid beads in a deep freezer also led to a 30% decrease in ammonia removal and urea secretion rates compared to the group cryopreserved in liquid nitrogen, regardless of the cryopreservation period.

In this study, albumin synthesis of cultured hepatocyte spheroid and spheroid beads was particularly low after thawing. Hepatocytes are easily damaged by the freeze–thaw process^11^. Several studies have specifically discussed cryopreservation of animal hepatocytes for application in BAL. For instance, Nyberg et al.^12^ compared albumin production per cell, and the albumin production of cryopreserved cells was 2% of that of fresh cells. The results of albumin synthesis analysis in this study are consistent with those of other past researchers^13–15^.

During the process of freezing cells, latent heat of fusion occurs at the point of freezing, causing ice crystals to form and melt, which then causes cell destruction. The freezing rate has a significant impact on cell damage^16^. To minimize cell damage, slow freezing protocols are considered the best strategy for cryopreserving hepatocytes. When the crystallization begins, the latent heat of fusion is suddenly released, and the cell sample becomes warm. As this heat release can be detrimental, we developed a slow linear freezing protocol (− 1 °C/min) to minimize warming by introducing a shock-cooling step.

The result of BAL operation (BLN, BDF BAL) for 24 h after freeze/thaw of spheroids beads in large quantities revealed lower liver function activity than the result of BAL operation (SLNB, SDFB BAL) by immobilizing spheroids after freeze/thaw. The results of the SDFB BAL operation after cryopreservation in a deep freezer for 2 months showed no statistically significant difference in ammonia removal or urea secretion up to 10 h compared to the results of the SLNB BAL operation. However, differences in liver function activity emerged beyond 10 h.

Unlike the cryopreservation of spheroids using vials, operating BAL for approximately 10 h resulted in similar liver function activity with liquid nitrogen and deep freezer cryopreservation. However, liver function activity exhibited a difference after 10 h. This suggests that deep freezer cryopreservation is also useful, considering the issue of transporting frozen hepatocyte spheroids and the assumption that BAL operation lasts only 10 h.

The frozen hepatocyte spheroids cells concentration used in the 1/10 scale BAL system using a commercially available bag was 1.5 × 10^9^ cells/75 mL/bag. For a clinical BAL system with 3 × 10^10^ cells, approximately 20 bags are required. Cryopreserving hepatocyte spheroid beads for a 1/10 scale BAL system requires a cell concentration of 7.5 × 10^8^ cells/75 mL/bag, translating to approximately 40 bags for a clinical BAL system. To address this, the research team improved the freezing bag to accommodate a mass system, increasing the cryopreservation capacity per bag to 250 mL. This enhancement allows for the cryopreservation of 7.5 × 10^9^ cells/250 mL/bag for hepatocyte spheroids, enabling the cryopreservation of 3 × 10^10^ cells with a total of 4 bags. Even for hepatocyte spheroid hydrogel beads, a concentration of 3.75 × 10^9^ cells/250 mL/bag is achievable, facilitating the cryopreservation of 3 × 10^10^ hepatocytes filled in a reactor with a total of 8 bags. Solutions containing 3 × 10^10^ cryopreserved hepatocyte spheroids would have a volume of 1L, and an additional 30 L of thawing medium (at a 1:30 ratio) would be required to properly dilute the cryopreservation reagent (dimethyl sulfoxide [DMSO]). Moreover, 3 × 10^10^ hepatocyte spheroid beads would constitute a volume of 2L, and 60 L of thawing medium would be required for resuscitation^17^.

When hepatocyte spheroids are frozen/thawed to produce hydrogel beads for producing a BAL system, 4 freezing bags and approximately 30 L of thawing medium will be required and approximately 20 h can be saved. However, if cryopreserved spheroid hydrogel beads are applied to the BAL system, 8 freezing bags and approximately 60 L of thawing medium will be required and approximately 26 h can saved while constructing the BAL system. In the SLNB, SDFB, and BLN groups, the viabilities of spheroid beads filled in the bioreactor of the BAL system before operation were similar (Fig. 6B); however, liver function activity during the 24-h BAL system operation was significantly higher in the SLNB group compared with the other groups. In conclusion, manufacturing beads after the cryopreservation of spheroids should be considered the best method in terms of efficiency, economy, and the liver function activity of the manufacturing process of the BAL system.

## Materials and methods

### Animals

Male pigs grown in clean barrier facilities (15–22 weeks old, mixed strain of Landrace, Yorkshire, and Duroc weighing 12–18 kg, Optipharm Co. Ltd. Cheongju, Korea) were used for hepatocyte isolation. This study was reviewed and approved by the Institutional Animal Care and Use Committee (IACUC) of the Samsung Biomedical Research Institute (SBRI) at Samsung Medical Center (SMC) (Approval No. 20180131002). All experiments were performed in accordance with the guidelines and regulations of the IACUC. The SBRI is a facility of the Accreditation of Laboratory Animal Care International (AAALAC International) and adheres to the Institute of Laboratory Animal Resources (ILAR) guidelines. The study followed the ARRIVE guidelines for reporting animal research^18^.

### Porcine hepatocyte isolation

Hepatocytes were harvested from 15- to 22-week-old male pigs weighing 12–18 kg. Ketamine (20 mg/kg intramuscularly) and xylazine (2 mg/kg intramuscularly) were administered to induce anesthesia. After endotracheal intubation, the ventilator was adjusted to achieve a PCO_2_ of 35–40 torr. Anesthesia was maintained with isoflurane (2%) and vecuronium (2 mg/kg/h). Hepatocytes were isolated using a three-step collagenase perfusion technique using a method described by Lee et al.^19^. First, the liver was perfused via the portal vein using 8 L of warm oxygenized perfusion buffer at a flow rate of 80 mL/min/kg of body weight. Subsequently, 1000 mL of a solution containing calcium was introduced via the portal vein to allow optimal collagenase function. Lastly, the liver was digested by recirculating 1500 mL of a perfusion buffer supplemented with collagenase (0.3 g/L) and calcium chloride (0.735 g/L) at a flow rate of 70 mL/min/kg body weight.

When the liver became softer and the capsule began to rupture spontaneously, the collagenase solution was immediately removed and the digestion process was inhibited by adding 1000 mL of cold (4 °C) Williams E medium (Sigma-Aldrich, St. Louis, MO, USA). The liver was then cooled in a sterile container on ice, minced, and gradually filtered through a mesh (pore size: 500–150 μm). The liver cells were centrifuged, the cell pellet was removed with medium, and cells were centrifuged thrice at 50×*g* for 4 min at 4 °C. Trypan blue exclusion staining revealed that more than 85% of hepatocytes were viable.

### Spheroid culture and calcium-alginate immobilization

Hormonally defined medium (HDM) comprising William’s E medium and supplemented with 20% albumin (5 mL/L), epidermal growth factor (20 μg/L), insulin (10 mg/L), CuSO_4_·5H_2_O (24.97 μg/L), ZnSO_4_·7H_2_O (14.38 pg/L), H_2_SeO_3_ (3 μg/L), NaHCO_3_ (1.05 g/L), HEPES (1.19 g/L), penicillin (58.8 mg/L), and streptomycin (0.1 g/L) was used to culture spheroids. Spheroid culturing and calcium alginate immobilization were performed using a previously described protocol^19^. First, the isolated hepatocytes were inoculated at a cell density of 1.5 × 10^6^ cells/mL in a spinner flask containing 2000 mL of culture medium and stirred with a magnetic stirrer at 20 rpm. The gas phase of the spinner flask was purged with 95% O_2_ and 5% CO_2_ to provide the high oxygen demand of hepatocytes. These cells were then cultured for 10 h to form spheroids with a diameter of 75 ± 28 μm. Cell viability was assessed by performing trypan blue dye exclusion and 3-(4, 5-dimethyl thiazol-2-yl)-2, 5-diphenyltetrazolium bromide (MTT) conversion before encapsulation. Hepatocyte spheroids were mixed with a 1.5% alginate solution, which had been previously heat-treated to allow dissolution and sterilization. The mixture was subsequently kept in a high content/speed immobilization apparatus and dropped into a 100 mM calcium solution.

### Cryopreservation and thawing

The freezing procedure was performed under an ice bath. Hepatocyte spheroids or immobilized hepatocyte spheroid beads were centrifuged at 50×*g* for 5 min at 4 °C. The supernatant was discarded, and the hepatocyte spheroids and immobilized hepatocyte spheroid beads were resuspended in ice-cold cryopreservation solution based on the protocol given by Lee et al.^17^ using modified histidine-tryptophan-ketoglutarate (mHTK; containing 4% human serum albumin, 3000 U/L insulin, 50 mg/L dexamethasone, and 0.04% amino acids) with N-acetylcysteine (15 mM NAC). The cryopreservation solution contained 15% dimethyl sulfoxide (DMSO) as a cryoprotectant.

Cell solution at a density of 1 × 10^7^ cells/mL was transferred to cryovials. These vials were then quickly placed into an isopropanol progressive freezing container at − 80 °C overnight and immersed in liquid nitrogen after 24 h. Cryopreserved hepatocyte spheroids and immobilized spheroids beads were rapidly thawed at 37 °C in a water bath one month and one year later, respectively. The thawed cell suspension was transferred to a tube containing 30 mL of the warmed (37 °C) thawing medium (washing medium supplemented with 10% FBS) and mixed. The resulting cell suspension was centrifuged at 50×*g* for 5 min at 4 °C. The supernatant was removed and the cell pellet was resuspended in HDM. Hepatocyte spheroids were plated in a collagen-coated plastic dish (5 × 10^5^ cells/mL) and cultured for 24 h to perform functional assays. For subsequent analyses, hepatocyte spheroid beads were resuspended in HDM at a cell density of 5 × 10^5^ cells/mL in plastic dishes.

Hepatocyte spheroids and immobilized spheroids beads were cryopreserved using a freezing bag to use in the BAL system with a 1/10 scale-down bioreactor. The solution of hepatocyte spheroids (1.5 × 10^9^ cells/75 mL/bag) was transferred to a freezing bag (CryoMACS Freezing Bag 250). Subsequently, approximately 25 mL of immobilized hepatocyte spheroid beads (3 × 10^7^ cells/mL) were added to 50 mL of cryopreservation solution (mHTK + NAC) containing 22.5% (v/v) DMSO to obtain a final DMSO concentration of 15%, and the solution with beads was transferred to the freezing bag.

These freezing bags were then quickly placed into a controlled rate freezer (CRF, CryoMed, Thermo Forma, Marietta, OH, USA). The freezing process was performed using an optimized cooling program modified reported by Lee et al.,^20^ which included the following steps: wait at 4 °C, decrease at a rate of − 1 °C/min to − 20 °C, − 50 °C/min to − 70 °C, hold for 2 min at − 70 °C, decrease at a rate of − 10 °C/min to − 40 °C, − decrease at a rate of 1 °C/min to − 80 °C, and finally decrease at a rate of and − 2 °C/min to − 160 °C. The temperature in the cell suspension was measured continuously using the temperature probe of the machine. When the freezing process was completed, the freezing bags were quickly transferred in a vapor phase of liquid nitrogen or a deep freezer at − 80 °C.

Cryopreserved hepatocyte spheroids and immobilized hepatocyte spheroids beads were obtained after 2 months by rapidly thawing in a water bath at 40 °C. Cells were collected and 30 times the volume of HDM containing 10% FBS at 37 °C was added and mixed gently. These cells were then washed twice to remove the cryoprotectant by centrifugation for 4 min at 50×*g* at 4 °C. Hepatocyte spheroids were then immobilized using calcium alginate. Immobilized hepatocyte spheroids beads were used to pack the bioreactor.

### Bioreactor and BAL system

**Figure 7:**
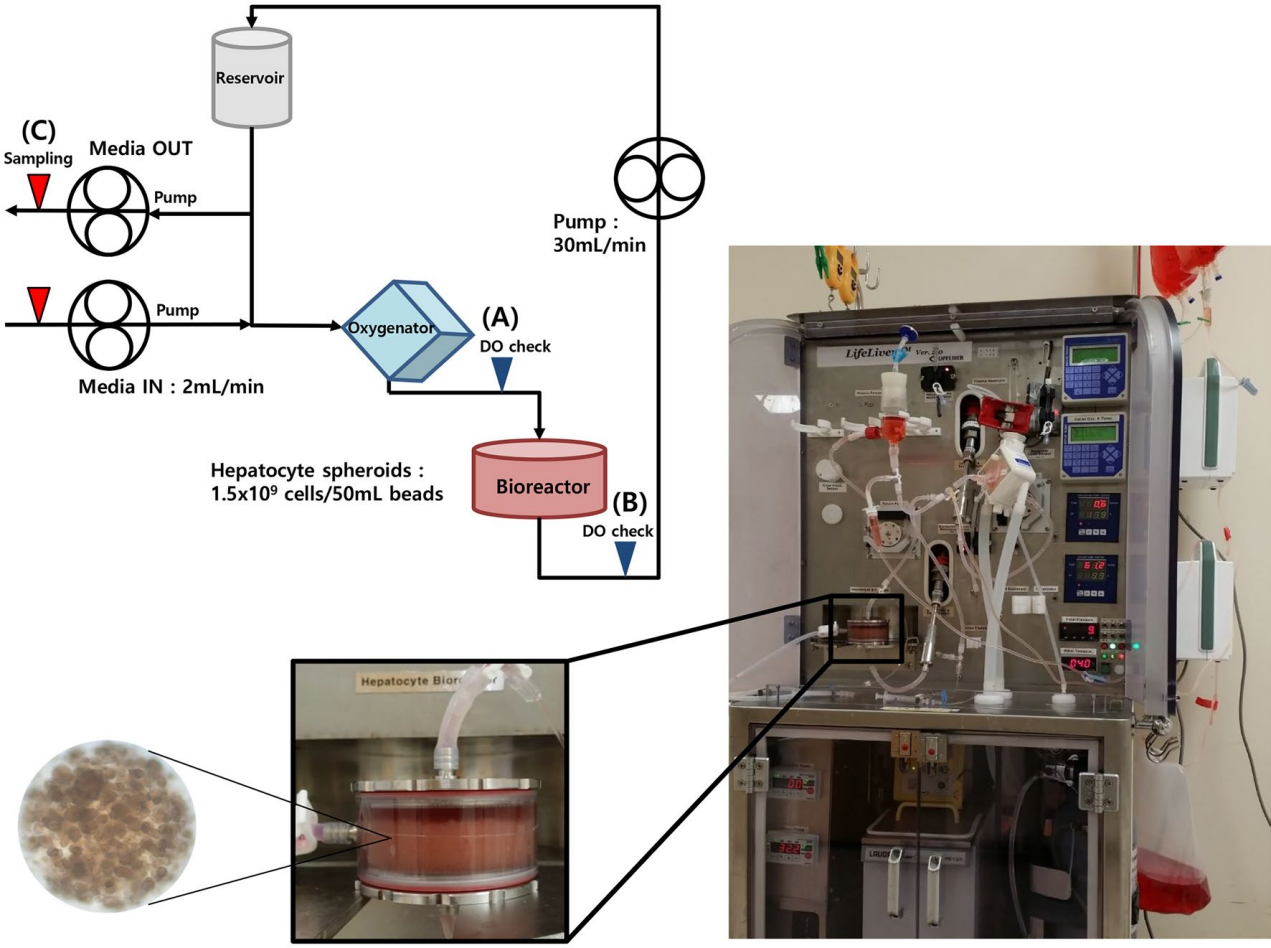
Schematic flow diagram of a bioartificial liver (BAL) operating system. A new medium that flows into the system (Media IN) is later removed (Media OUT). The medium passes through the BAL system and recirculates at 30 mL/min through the reservoir, oxygenator/heater, and bioreactor containing hepatocyte spheroids hydrogel beads. The samples used for measuring dissolved oxygen were collected at (**A**) and (**B**), while the samples for biochemical analyses were collected at (**C**).

The BAL system used in the *in-vitro* study was a 1/10 scaled-down version of the previously developed BAL system described by Lee et al.^19^. The BAL system contained a medium reservoir, oxygenator, pump, and a cylindrical-type calcium-alginate packed-bed bioreactor containing immobilized hepatocyte spheroids. The medium enters the BAL system at a rate of 2 mL/min and recirculates at a rate of 30 mL/min through the reservoir, oxygenator/heater, and bioreactor. After recirculating through the BAL system, the medium is removed. The total cell number in the BAL system was 1.5 × 10^9^, which was seeded in a bead solution of 50 mL, as shown in the schematic diagram of the BAL operating system (Fig. 7).

### Experimental design for comparing hepatocyte cryopreservation processes

**Figure 8:**
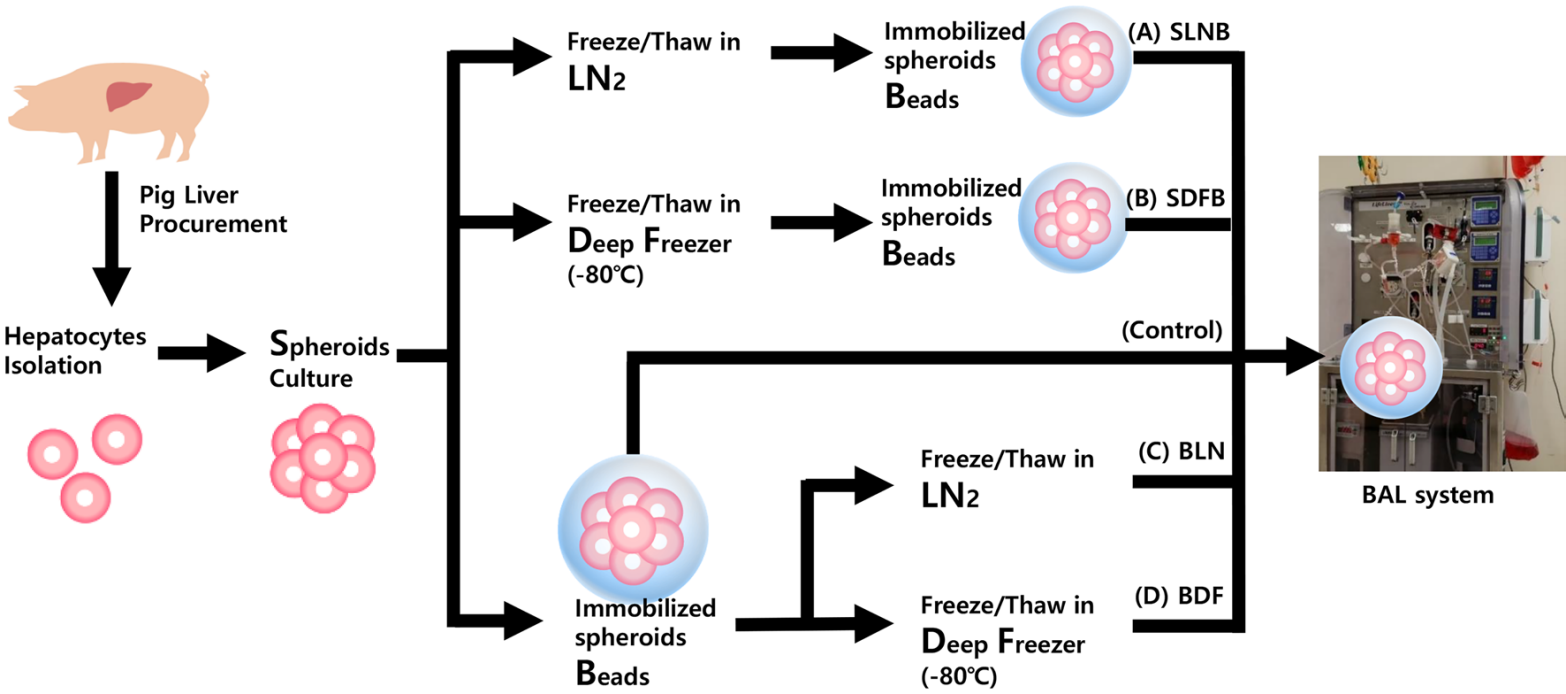
Schematic flow diagram displaying the bioaritificial liver (BAL) system manufacturing process and study groups: (**A**) LN_2_ freeze/thaw–spheroids immobilization on hydrogel beads, (SLNB) group, (**B**) Deep freezer freeze/thaw–spheroids immobilization on hydrogel beads (SDFB) group, (**C**) Spheroids immobilization on hydrogel beads–LN_2_ freeze/thaw (BLN) group, (**D**) Spheroids immobilization on hydrogel beads–deep freezer freeze/thaw (BDF) group, and spheroids immobilization on hydrogel beads without freeze/thaw (Control).

This study included the following five experimental groups: (a) SLNB group, (b) SDFB group, (c) BLN group, (d) BDF group, and (e) control group. In the control group, immobilized hepatocyte spheroids beads without cryopreservation were used in the BAL system for 24 h. In the SLNB group, cryopreserved hepatocyte spheroids in LN2 were thawed immobilized, and subsequently used in the BAL system. In the SDFB group, cryopreserved hepatocyte spheroids kept at − 80 °C (in a deep freezer) were thawed and immobilized, then used in the BAL system. In the BLN group, immobilized hepatocyte spheroids beads cryopreserved in LN2 were thawed and subsequently used in the BAL system. In the BDF group, immobilized hepatocyte spheroids beads that were cryopreserved at − 80 °C (in a deep freezer) were thawed and subsequently used in the BAL system. Figure 8 illustrates the complete BAL manufacturing process and the study group exposed to different freeze/thaw conditions.

### Cell viability assays

The viability of thawed hepatocytes was determined using the trypan blue dye exclusion methods. The viability and attachment of thawed hepatocyte spheroids were determined by using an MTT conversion assay. To demonstrate the viability of cells in different cryopreservation/thawing solutions, calcium-alginate beads containing hepatocyte spheroids in the experimental and control groups were assessed using the MTT assay. The Live/Dead viability/cytotoxicity kit from Molecular Probes, Inc. (Eugene, OR, USA) was used to assess live/dead staining in each sample according to the manufacturer’s instructions. Live cells were stained with calcein-AM, which is metabolically converted by intracellular esterases into calcein and a green fluorescent product, while the dead cells were stained with ethidium bromide. The cells were incubated in a medium containing these reagents for 10–15 min at 37 °C, after which they were immediately imaged using an Olympus IX71 instrument (Olympus Co., Tokyo, Japan).

### Biochemical analysis of hepatocyte functions

The rates of ammonia removal and urea production were evaluated to perform in vitro experiments using a previously described protocol^21^. To determine the rate of ammonia removal, the medium was supplemented with 0.3 mM of NH_4_Cl and circulated. Samples were collected every 2 h.

Of note, 640 µL of sodium tungstate (100 g/L) and 160 µL of the sample were added to a test tube and mixed with 1.0 mL of color reagent I containing the following ingredients: phenol (10 g/L), sodium nitroprusside (50 mg/L), and 1.0 mL of color reagent II containing NaOH (5 g/L), Na_2_HPO_4_·12H_2_O (53.6 g/L), and 1% sodium hypochlorite. After gentle vortex, tubes were kept at 37 °C for 20 min. The absorbance of the samples was detected at 630 nm. To determine the rate of urea production, test tubes were filled with 3 mL of a color reagent produced by mixing 20 mL of diacetyl monoxime (6 g/L) and thiosemicarbazide (0.3 g/L) and 100 mL of 34% H_3_PO_4_, followed by adding 100 µL of the sample. Subsequently, after gentle mixing, tubes were incubated at 100 °C for 10 min. The absorbance of the samples was detected at 540 nm.

Albumin concentration was determined using a pig albumin ELISA quantitation kit (E100–110; Bethyl Laboratories, Montgomery, TX, USA). Results were analyzed using the SoftMax Pro 4.8 software (Molecular Devices LLC, San Jose, CA, USA) and fitted with a 4-parameter curve at an R^2^ value of > 0.99.

### Statistical analysis

The GraphPad Prism® 5 software (GraphPad, San Diego, CA, USA) was used to perform statistical analysis. Data from at least four independent experiments are shown as mean ± standard deviation. Student’s *t*-test was performed to compare two independent groups, and comparisons between three or more groups with one independent variable were performed using one-way analysis of variance (ANOVA). A two-way repeated measurement ANOVA and Bonferroni’s post multiple comparisons tests were performed to compare groups exposed to different freeze/thaw conditions. A p-value of < 0.05 was considered statistically significant.

## Data Availability

The datasets used and/or analysed during the current study are available from the corresponding author on reasonable request.

## References

[1] Bernal W, Auzinger G, Dhawan A, Wendon J (2010). Acute liver failure. Lancet.

[2] Lee WM (2012). Recent developments in acute liver failure. Best Pract. Res. Clin. Gastroenterol..

[3] Heydari Z, Najimi M, Mirzaei H, Shpichka A, Ruoss M, Farzaneh Z, Montazeri L, Piryaei A, Timashev P, Gramignoli R (2020). Tissue engineering in liver regenerative medicine: Insights into novel translational technologies. Cells.

[4] Strain AJ, Neuberger JM (2002). A bioartificial liver–state of the art. Science.

[5] Thompson J, Jones N, Al-Khafaji A (2018). Extracorporeal cellular therapy (ELAD) in severe alcoholic hepatitis: A multinational, prospective, controlled, randomized trial. Liver Transpl..

[6] van Wenum M, Treskes P, Adam AAA, van der Mark VA, Jongejan A, Moerland PD (2018). HepaRG-progenitor cell derived hepatocytes cultured in bioartificial livers are protected from healthy- and acute liver failure-plasma induced toxicity. Cell Physiol. Biochem..

[7] Chen S, Wang J, Ren H (2020). Hepatic spheroids derived from human induced pluripotent stem cells in bio-artificial liver rescue porcine acute liver failure. Cell Res..

[8] Woods EJ, Thirumala S, Badhe-Buchanan SS, Clarke D, Mathew AJ (2016). Off the shelf cellular therapeutics: Factors to consider during cryopreservation and storage of human cells for clinical use. Cytotherapy.

[9] Massie I, Selden C, Hodgson H, Fuller B (2013). Storage temperatures for cold-chain delivery in cell therapy: A study of alginate-encapsulated liver cell spheroids stored at -80 degrees c or -170 degrees c for up to 1 year. Tissue Eng. Part C Methods.

[10] Kilbride P (2016). Impact of storage at -80 degrees C on encapsulated liver spheroids after liquid nitrogen storage. Bioresour. Open Access.

[11] Dou M, de Sousa G, Lacarelle B, Placidi M, de La Porte P, Domingo M, Lafont H, Rahmani R (1992). Thawed human hepatocytes in primary culture. Cryobiology.

[12] Nyberg SL, Hardin J, Amiot B, Argikar UA, Remmel RP, Rinaldo P (2005). Rapid, large-scale formation of porcine hepatocyte spheroids in a novel spheroid reservoir bioartificial liver. Liver Transplant..

[13] Mahler S, Desille M, Frémond B, Chesné C, Guillouzo A, Campion J-P, Clément B (2003). Hypothermic storage and cryopreservation of hepatocytes: The protective effect of alginate gel against cell damages. Cell Transplant..

[14] Aoki T, Koizumi T, Kobayashi Y, Yasuda D, Izumida Y, Jin Z, Nishino N, Shimizu Y, Kato H, Murai N (2005). A novel method of cryopreservation of rat and human hepatocytes by using encapsulation technique and possible use for cell transplantation. Cell Transplant..

[15] Kusano T, Aoki T, Yasuda D, Matsumoto S, Jin Z, Nishino N, Hayashi K, Odaira M, Yamada K, Koizumi T (2008). Microencapsule technique protects hepatocytes from cryoinjury. Hepatol. Res..

[16] Hengstler JG (2001). Cryopreserved primary hepatocytes as a constantly available in vitro model for the evaluation of human and animal drug metabolism and enzyme induction. Drug Metab. Rev..

[17] Lee JH (2022). Establishment of a serum-free hepatocyte cryopreservation process for the development of an "Off-the-Shelf" bioartificial liver system. Bioengineering.

[18] Kilkenny C, Browne WJ, Cuthill IC, Emerson M, Altman DG (2010). Improving bioscience research reporting: The ARRIVE guidelines for reporting animal research. PLoS Biol..

[19] Lee JH (2017). Functional evaluation of a bioartificial liver support system using immobilized hepatocyte spheroids in a porcine model of acute liver failure. Sci. Rep..

[20] Lee JH, Jung DH, Lee DH, Park JK, Lee SK (2012). Slow cooling rate with a shock cooling program can effectively cryopreserve pig hepatocytes. Transplant. Proc..

[21] Lee JH, Lee DH, Son JH, Park JK, Kim SK (2005). Optimization of chitosan-alginate encapsulation process using pig hepatocytes for development of bioartificial liver. J. Microbiol. Biotechnol..

